# Hypothesized diprotomeric enzyme complex supported by stochastic modelling of palytoxin-induced Na/K pump channels

**DOI:** 10.1098/rsos.172155

**Published:** 2018-03-21

**Authors:** Gabriel D. Vilallonga, Antônio-Carlos G. de Almeida, Kelison T. Ribeiro, Sergio V. A. Campos, Antônio M. Rodrigues

**Affiliations:** 1 Department of Computer Science, Universidad Nacional de San Luis, San Luis, Argentina; 2 Department of Biosystems Engineering, Federal University of São João del Rei, São João del Rei, Minas Gerais, Brazil; 3 Department of Computer Science, Federal University of Minas Gerais, Belo Horizonte, Minas Gerais, Brazil

**Keywords:** palytoxin, Na/K pump, diprotomer, probabilistic model, statistical model checker

## Abstract

The sodium–potassium pump (Na^+^/K^+^ pump) is crucial for cell physiology. Despite great advances in the understanding of this ionic pumping system, its mechanism is not completely understood. We propose the use of a statistical model checker to investigate palytoxin (PTX)-induced Na^+^/K^+^ pump channels. We modelled a system of reactions representing transitions between the conformational substates of the channel with parameters, concentrations of the substates and reaction rates extracted from simulations reported in the literature, based on electrophysiological recordings in a whole-cell configuration. The model was implemented using the UPPAAL-SMC platform. Comparing simulations and probabilistic queries from stochastic system semantics with experimental data, it was possible to propose additional reactions to reproduce the single-channel dynamic. The probabilistic analyses and simulations suggest that the PTX-induced Na^+^/K^+^ pump channel functions as a diprotomeric complex in which protein–protein interactions increase the affinity of the Na^+^/K^+^ pump for PTX.

## 1. Introduction

The mechanism for moving sodium and potassium ions through the cell membrane (sodium out and potassium into the cell, both against their concentration gradients) is an active transport process involving the hydrolysis of adenosine triphosphate (ATP). This mechanism uses an enzyme called Na^+^/K^+^-ATPase that accomplishes the transport of three Na^+^ molecules and two K^+^. This pumping system is crucial for cell physiology, particularly in neurons [1]. Despite great advances in the understanding of this ionic pumping system, it is not completely understood.

Currently, the Albers–Post model is the most acceptable model for explaining how Na^+^/K^+^-ATPase exchanges Na^+^ and K^+^ and it describes the Na^+^/K^+^ pump in terms of alternating gates[2–4]. Supporting this model, experiments performed with palytoxin (PTX) are used to uncouple the alternating gates, allowing them to open simultaneously. Therefore, the PTX–Na^+^/K^+^ pump complex forms a non-selective monovalent ionic channel. Investigation of this channel has provided recent advances in the understanding of the Na^+^/K^+^ pump [4–6].

Recently, a reaction model was proposed to investigate the kinetic mechanisms of the phosphorylation and dephosphorylation of the PTX–Na^+^/K^+^ pump complex [7]. Using this model, experimental procedures were simulated [6,7] to investigate reactions and substates responsible for increasing the enzyme affinity for PTX and its effects on enzyme phosphorylation [8]. A reaction model was also proposed to investigate the interference of PTX with the pump when the enzyme interacts with Na^+^ and/or K^+^ in the absence of ATP [9]. These models resulted in the proposal of a set of reactions that lead to substates of the PTX–Na^+^/K^+^ pump complex, following reported descriptions [2,4–6]. Simulations performed with these models reproduced experimental recordings, based on the calculation of the PTX-induced current and the permeability of the formed channels to monovalent ions in terms of the concentration of the substate PTXE. This approach allowed for investigating the substates of the PTX–pump complex, taking into account whole-cell ionic current recordings; however, the substates of the formed single channels are not accessible.

In this work, we used a statistical model checker to investigate single channels induced by PTX. The substates and reaction rates extracted from the literature [9] were represented mathematically. Using probabilistic analyses and simulating populations of single channels based on stochastic semantics, the model was validated and the single-channel dynamic was compared with experimental data. From this comparison, the inclusion of additional substates was deduced, considering that the Na^+^/K^+^ pump functions as a diprotomeric complex. The simulation also allowed proposing that the protein–protein interactions of the diprotomer increase the affinity of the pump for PTX and can therefore enhance its toxic effect even when the toxin concentration is low.

## 2. Material and methods

In this work, we investigate the PTX–pump channel formed in the presence of Na^+^ and absence of ATP according to experimental recordings [5,6]. This is a condition where the pump may still bind Na^+^ exhibiting changes to different states; however, the normal cycle of ionic transport does not occur, and this situation represents an important reduction in the number of reactions and substates for the Na^+^/K^+^-ATPase. Two groups of reactions were taken into account to model the PTX–Na^+^/K^+^ pump complex [9]: (i) reactions extracted from the Albers–Post model for the pump (table 1) and (ii) reactions representing interactions between PTX and the pump (table 2). These reactions do not describe the complete cycle for the transport of Na^+^ and K^+^ through the pump. They describe only the dynamic of the pump in the presence of Na^+^ and absence of the other ligands.

**Table 1.** Reactions and substates of the Na^+^/K^+^-ATPase considering only Na^+^ as the sole physiological ligand present, extracted from the Albers–Post model.

index	reaction	reaction rate
1	E2 ↔E1	$r_{1}=\alpha_{1}[E2]-\beta_{1}[E1]$
2	$3Na^{+,i}+E1↔{\mathrm{Na}}_{3}^{+}E1$	$r_{2}=\alpha_{2}\frac{{\left({\left[Na^{+}\right]}^{i}\right)}^{3}}{{\left({\left[Na^{+}\right]}^{i}+Kd_{\mathrm{Na}}\right)}^{2}}[E1]-\beta_{2}[{\mathrm{Na}}_{3}^{+}E1]$
3	(Na^+^)_2_E2 ↔ E1 + 2Na^+,i^	$r_{3}=\alpha_{3}[{\left({\mathrm{Na}}^{+}\right)}_{2}E2]-\beta_{3}\frac{{\left({\left[Na^{+}\right]}^{o}\right)}^{2}}{{\left({\left[Na^{+}\right]}^{o}+Kd_{\mathrm{Na}}\right)}^{2}}[E1]$

**Table 2.** Reactions for the PTX–pump complex model (from Rodrigues *et al*. [9]).

index	reaction	reaction rate
p1	PTX^o^ + E1 ↔ PTXE_c_	$r_{p1}=\alpha_{p1}{\left[\mathrm{PTX}\right]}^{o}[E1]-\beta_{p1}[E1]$
p2	PTX^o^ + E2 ↔PTXE_c_	$r_{p2}=\alpha_{p2}{\left[\mathrm{PTX}\right]}^{o}[E2]-\beta_{p2}[\mathrm{PTX}E_{c}]$
p3	$\mathrm{PT}X^{o}+{\mathrm{Na}}_{3}^{+}E1↔\mathrm{PTX}E_{c}+3Na^{+,i}$	$r_{p3}=\alpha_{p3}{\left[\mathrm{PTX}\right]}^{o}[{\mathrm{Na}}_{3}^{+}E1]-\beta_{p3}[\mathrm{PTX}E_{c}]\frac{{\left({\left[Na^{+}\right]}^{i}\right)}^{3}}{{\left({\left[Na^{+}\right]}^{i}+K_{\mathrm{dNa}}\right)}^{2}}$
p4	$2Na^{+,o}+\mathrm{PTXE}↔\mathrm{PTX}(Na^{+}{)}_{2}E_{c}$	$r_{p4}=\alpha_{p4}\frac{{\left({\left[Na^{+}\right]}^{o}\right)}^{2}}{{\left({\left[Na^{+}\right]}^{o}+K_{\mathrm{dNa}}\right)}^{2}}[\mathrm{PTXE}]-\beta_{p4}[\mathrm{PTX}(Na^{+}{)}_{2}E_{c}]$
p5	$\mathrm{PTX}(Na^{+}{)}_{2}E_{c}↔\mathrm{PTX}E_{o}+2Na^{+,i}$	$r_{p5}=\alpha_{p5}[\mathrm{PTX}(Na^{+}{)}_{2}E_{c}]-\beta_{p5}\frac{{\left({\left[Na^{+}\right]}^{i}\right)}^{2}}{{\left({\left[Na^{+}\right]}^{i}+K_{\mathrm{dNa}}\right)}^{2}}[\mathrm{PTX}E_{o}]$
p6	$\mathrm{PTX}(Na^{+}{)}_{2}E_{c}↔{\left(Na^{+}\right)}_{2}E2+\mathrm{PT}X^{o}$	$r_{p6}=\alpha_{p6}[\mathrm{PTX}(Na^{+}{)}_{2}E_{c}]-\beta_{p6}[{\left(Na^{+}\right)}_{2}E2]{\left[\mathrm{PTX}\right]}^{o}$

**Figure 1.**
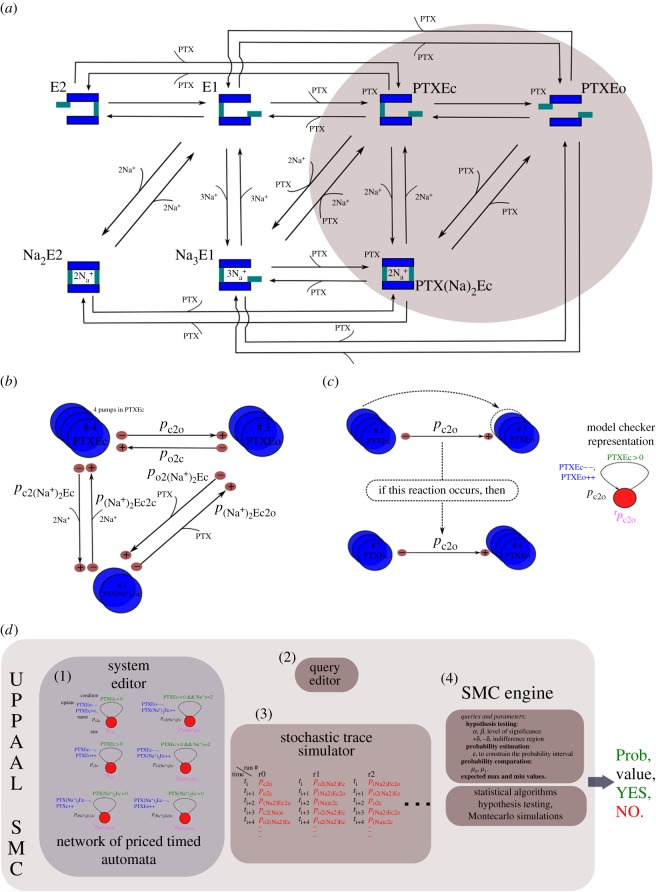
Statistical model of the PTX–Na^+^/K^+^ pump complex. (*a*) Diagram of the reactions and substates for the PTX–pump complex model. (*b*) General principle of the statistical model. (*c*) One of the four enzyme units in the substate PTXEc transitioning to the substate PTXEo, according to one of the directions of reaction p8. (*d*) Network of stochastic hybrid automata used to model the six possible transitions between the substates indicated in (*b*).

The probabilistic model of the PTX–Na^+^/K^+^ pump complex and the general characteristics of the model developed in UPPAAL-SMC [10,11] were based on the representation of the reactions in tables 1 and 2, with additional reactions shown in table 3, which describe transitions that lead to the substate of the PTX–pump channel being opened. In figure 1, a diagrammatic representation of all transitions between the substates of the model is shown. The simulations and the probabilistic queries performed by the statistical model checker are generated according to stochastic semantics [10,11].

**Table 3.** Reactions for the PTX–pump complex model to describe single-channel activity.

index	reaction	reaction rate
p7	E1 + PTX^o^ ↔ PTXE_o_	$r_{p7}=\alpha_{p7}{\left[\mathrm{PTX}\right]}^{o}[E1]-\beta_{p7}[\mathrm{PTX}E_{o}]$
p8	PTXE_c_ ↔PTXE_o_	$r_{p8}=\alpha_{p8}[\mathrm{PTX}E_{c}]-\beta_{p8}[\mathrm{PTX}E_{o}]$
p9	$2Na^{+,o}+\mathrm{PTX}E_{o}↔\mathrm{PTX}(Na^{+}{)}_{2}E_{c}$	$r_{p9}=\alpha_{p9}\frac{{\left({\left[Na^{+}\right]}^{o}\right)}^{2}}{{\left({\left[Na^{+}\right]}^{o}+K_{\mathrm{dNa}}\right)}^{2}}[\mathrm{PTX}E_{o}]-\beta_{p9}[\mathrm{PTX}(Na^{+}{)}_{2}E]$
P10	${\mathrm{Na}}_{3}^{+}E1+\mathrm{PT}X^{o}↔3Na^{+,i}+\mathrm{PTX}E_{o}$	$r_{p10}=\alpha_{p10}[{\mathrm{Na}}_{3}^{+}E1]{\left[\mathrm{PTX}\right]}^{o}-\beta_{p10}\frac{{\left(N{a^{+}}^{i}\right)}^{3}}{{\left({\left[Na^{+}\right]}^{i}+K_{\mathrm{dNa}}\right)}^{3}}{\left[\mathrm{PTXE}\right]}^{o}$

To explain the general principle of the present model, we show in figure 1*b* three substates (PTXE_c_, PTXE_o_ and PTX(Na^+^)_2_E_c_) and a number of enzyme units in each of these substates: four in PTXE_c_, three in PTXE_o_ and two in PTX(Na^+^)_2_E_c_. In figure 1*c*, one of the four enzyme units in the substate PTXE_c_ is shown transitioning to the substate PTXE_o_, according to one of the directions of reaction p8. This transition is modelled as a continuous-time Markov chain process, a network of priced timed automata. The transition occurs if there are enough reactants, or enzymes units, in the substate PTXE_c_ (PTXE_c_ > 0) and must follow a kinetic law encoded as an exponential rate that defines the distribution of the delay for the transition based on the reaction rate (*p*_c2o_ = *r*_p8_). When the transition is performed, the number of enzyme units in the substate PTXE_c_ is decreased (PTXE_c_−−), and the number of enzyme units in the substate PTXE_o_ is increased (PTXE_o_++). Figure 1*d* shows the network of priced timed automata used to model the six possible transitions between the substates indicated in figure 1*b*.

Taking into account the number of enzymes in the substates representing the open channel, *n*_ICo_, the PTX-induced Na^+^ permeability was estimated by

2.1 $$P_{\mathrm{Na}}^{\mathrm{PTX}}=\gamma \cdot n_{\mathrm{ICo}},$$

where *γ* is a parameter representing the Na^+^ permeability through one channel. Using the Goldman–Hodgkin–Katz current equation, it is possible to estimate the total current induced by PTX:

2.2 $$J_{\mathrm{Na}}^{\mathrm{PTX}}=P_{\mathrm{Na}}^{\mathrm{PTX}}z_{\mathrm{Na}}^{2}\frac{F^{2}V_{m}}{RT}\frac{{\left[Na^{+}\right]}^{i}e^{\frac{z_{\mathrm{Na}}FV_{m}}{\mathrm{RT}}}-{\left[Na^{+}\right]}^{o}}{e^{\frac{z_{\mathrm{Na}}FV_{m}}{RT}}-1},$$

where *z*_Na_ is the Na^+^ valence, *V*_m_ the membrane potential, *F* the Faraday constant, *R* the gas constant, *T* the absolute temperature, and [Na^+^]^i^ and [Na^+^]^o^ the Na^+^ intra- and extracellular ionic concentrations, respectively.

For all simulations performed in this study, it was assumed that [Na^+^]^i^ = 150 mM and [Na^+^]^o^ = 160 mM. These are the same concentrations used in the reported experimental procedures and were also used in the computational simulations [5,6,9]. The initial conditions used were as follows: $n_{E1}=n_{\mathrm{total}},n_{E2}=\;n_{\mathrm{Na}3E1}=n_{E2}=n_{\mathrm{Na}2E2}=n_{\mathrm{PTXEc}}=n_{\mathrm{PTXEo}}=n_{\mathrm{PTX}(\mathrm{Na})2\mathrm{Ec}}=0$. An epoch of at least 10 s of duration was simulated in the absence of PTX to equilibrate the system. Afterwards, the PTX concentration was modified in accordance with the simulated experimental protocols. The time increment used in the simulations was 0.01 s.

The rate constants of the reactions described in tables 1 and 2 were extracted from published data (table 4). The constants of reaction 1 were extracted from an experimental report where the authors investigated the pump cycle when only Na^+^ is transported [12]. The constants of reactions 2 and 3 were based on values obtained from a detailed description of Na^+^ and K^+^ binding to the Na/K-ATPase during the complete transport cycle [13]. The reaction constants of p1–p6 were extracted from Rodrigues *et al*. [9]. The reaction rates of p7, p9 and p10 (table 3) were assumed to be equal to the rates of reactions p1, p4 and p3, respectively (table 2). The constants of reaction p8 (*α*_p8_ and *β*_p8_) were adjusted using probability estimation queries, aimed at simulating an isolated ionic channel with an opening probability in the experimentally estimated range [5,6].

**Table 4.** Rate constants of the Albers–Post and PTX–pump complex models.

		constants	
index	reference	*α_j_*	*β_j_*
1	Campos & Beaugé [12]	1.00 × 10^2^ s^−1^	1.00 × 10^−2^ s^−1^
2	Heyse *et al.* [13]	2.00 × 10^2^ mM^−1^ s^−1^	8.00 × 10^2^ s^−1^
3	Heyse *et al.* [13]	1.00 × 10^−1^ s^−1^	5.00 × 10^1^ s^−1^
p1	Rodrigues *et al.* [9]	3.23 × 10^3^ mM^−1^ s^−1^	1.66 × 10^−5^ s^−1^
p2	Rodrigues *et al.* [9]	4.30 × 10^2^ mM^−1^ s^−1^	2.20 × 10^−10^ s^−1^
p3	Rodrigues *et al.* [9]	5.15 × 10^2^ mM^−1^ s^−1^	6.60 × 10^−7^ mM^−1^ s^−1^
p4	Rodrigues *et al.* [9]	1.93 × 10^−4^ s^−1^	2.50 × 10^−4^ s^−1^
p5	Rodrigues *et al.* [9]	1.70 × 10^−4^ s^−1^	1.31 × 10^−4^ s^−1^
p6	Rodrigues *et al.* [9]	8.80 × 10^−5^ s^−1^	2.65 × 10^1^ mM^−1^ s^−1^

## 3. Results

**Figure 2.**
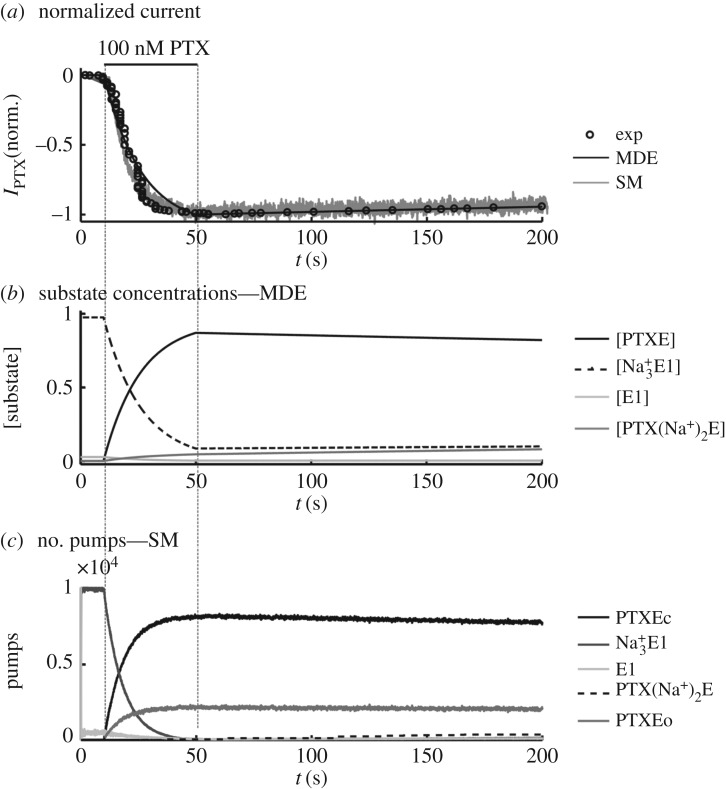
Activation of the PTX-induced current. (*a*) Current simulated with the SM. Grey curve (simulation) is compared with the normalized experimental data (circles—extracted from Artigas & Gadsby [6]) and with the simulation performed with the model of differential equations (MDE; black curve—extracted from Rodrigues *et al.* [9]). (*b*) Simulation performed with the MDE. The concentrations of the substates responsible for the induced current are shown. (*c*) Number of pumps occupying the different substates of the PTX–pump complex (as shown in (*b*), the substates are responsible for the induced current).

The activation and decay of the PTX-induced current in the presence of Na^+^ on both sides of the membrane were experimentally investigated [5,6], as shown in figure 2*a* (circles). The PTX application caused current activation through the channels induced in the Na^+^/K^+^-ATPase. This current reaches its maximum after 40 s. After discontinuation of perfusion with PTX, the current decay is slow, indicating a high affinity of the toxin towards the enzyme. Using a differential equation model, to describe the Na^+^/K^+^-ATPase reactions with the ligands, the experimental procedures were simulated [9], as shown in figure 2*a* (black line). The simulations allowed for proposing the dynamic of the Na^+^/K^+^ pump substates in the presence of the toxin (figure 2*b*). As shown, the PTX binding to the pump causes the transition to the substate PTXE. In this substate, the two pump gates may simultaneously open, characterizing the induced channel, and therefore the current activation. To characterize the channel opening, the authors assumed that the ionic permeability of the channel is proportional to the concentration of enzymes in the PTXE substate. For this calculation, the proportionality constant relates to the opening probability of the channel. Using the stochastic model (SM) proposed in this work, simulation of the PTX (100 nM) perfusion caused the activation of the toxin-induced current, as shown in figure 2*a* (grey line). This current also reaches the maximum after 40 s, reproducing the experimental findings [5]. Moreover, after PTX perfusion, when [PTX]^o^ = 0, the current decay was also slow, characterizing the high affinity of the toxin towards the Na/K-ATPase. This simulation was based on the representation of 10^4^ pumps. The substate occupation (figure 2*c*) was similar to simulations performed with the model of differential equations (MDE) [9] (figure 2*b*). Before the PTX perfusion, the pumps mainly occupied the substates Na^+^_3_E1 and E1. When PTX was applied, the transition from the substates PTXEc (closed induced channel) to PTXEo (opened induced channel) occurred simultaneously with the current activation. Subsequently, when [PTX]^o^ = 0, the decay in the number of pumps in these two substates was slow due to the high affinity of the PTX towards the pump, characterizing the slow decay of the PTX-induced current.

**Figure 3.**
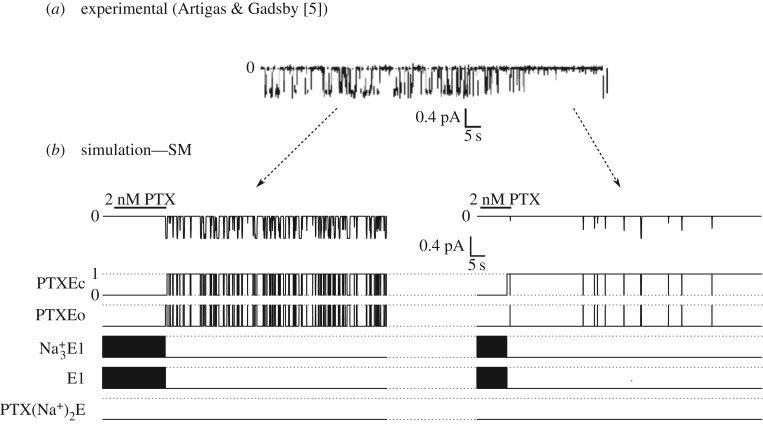
Single PTX-induced channel activity. (*a*) Perfusion with solution containing 2 nM of PTX caused the channel to open, showing epochs with intense open/close transitions and epochs where the induced channel remains in the closed state. (*b*) Simulation of the activity of the PTX-induced channel. Representing the experimental procedure, the [PTX]o was maintained equal to 2 nM up to the channel opening until the pump changed to the substate PTXEo. (Left) Simulation of the most intense period of activity, associated with transitions between the substates PTXEc and PTXEo. (Right) Simulation of period where the pump remains in the closed substate PTXEc most of the time.

The rate of reaction p8, adjusted during the simulations of the current activation (*α*_p8_ = 1.33 s^−1^ and *β*_p8_ = 5.09 s^−1^), allows for partially reproducing the experimentally recorded behaviour of the induced current of an isolated channel [5] (figure 3*a*). In the experiment, the authors perfused the Na/K-ATPase enzymes with an extracellular solution containing 2 nM PTX. The PTX removal was performed when the opening of a single channel was elicited. As the experiments showed, the induced channel remains active for a long period of time after the PTX washout. Moreover, the authors observed that after PTX removal, the induced channel exhibited continued gating followed by moments where the channel stays longer in the closed state. However, using the same reaction rates adjusted for p8, it was only possible to simulate the more intense activity of opening and closing after PTX removal, without the intermittent long periods of time in the closed state (figure 3*b*, left). Using the statistical tools available in the UPPAAL-SMC platform, it was verified that the probability of an enzyme remaining in the PTXEc substate, which would characterize a closed channel, is very low (between 0 and 9.73%) for dwell times greater than 7 s (table 5). For the adjustment of reaction 8, a high probability (between 90.20 and 100%) means the enzyme remains in the open state in intervals of less than 1 s and in the closed state in intervals shorter than 3 s, characterizing a state of intense activity when the channel is opening and closing.

**Table 5.** Pump probability to exhibit time remaining, *t*_p_, in the substates PTXEo and PTXEc, assuming the reaction rates of reaction p8 are constant (*α*_p8_ = 1.33 s^−1^ and *β*_p8_ = 5.09 s^−1^). Inferior and superior limit probability values are given for the pump in the substates PTXEo or PTXEc during the time interval indicated and the number of simulations necessary for the probability interval showing 95% significance.

	PTXEo			PTXEc		
		probability (%)			probability (%)	
interval for *t*_p_ (s)	number of simulations	inf	sup	number of simulations	inf	sup
(0, 1)	36	90.20	100	36	90.20	100
[1, 2)	398	40.26	50.26	36	90.20	100
[2, 3)	36	0	9.73	36	90.20	100
[3, 4)	36	0	9.73	171	83.19	93.17
[4, 5)	36	0	9.73	379	32.83	42.82
[5, 6)	36	0	9.73	150	5.19	15.16
[6, 7)	36	0	9.73	68	0.36	10.22
[7, 8)	36	0	9.73	36	0	9.73
[8, 9)	36	0	9.73	36	0	9.73
[9, 10)	36	0	9.73	36	0	9.73
..	..	..	..	..	..	..

To use the proposed model to simulate the channel staying in the closed state longer than in the opened state, it is necessary to readjust the constants of reaction p8 (*α*_p8_ = 1.33 × 10^−1^ s^−1^ and *β*_p8_ = 5.09 s^−1^) (figure 3*b*, right). For this adjustment, the probability of the enzyme remaining in the PTXEa state is also high (between 90.20 and 100%) in intervals of less than 1 s. However, there is a high probability that the enzyme remains longer (+10 s) in the PTXEc state (table 6). Therefore, although the channel can open, it has a high probability of remaining in the closed state longer.

**Table 6.** Pump probability to exhibit time remaining, *t*_p_, in the substrates PTXEo and PTXEc, assuming the reaction rates of reaction p8 are constant (*α*_p8_ = 1.33 × 10^−1^ s^−1^ and *β*_p8_ = 5.09 s^−1^). Inferior and superior limit probability values are given for the pump in the substates PTXEo or PTXEc during the time interval indicated and the number of simulations necessary for the probability interval showing 95% significance.

	PTXEo			PTXEc		
		probability (%)			probability (%)	
interval for *t*_p_ (s)	number of simulations	inf	sup	number of simulations	inf	sup
(0, 1)	36	90.2	100	36	90.20	100
[1, 2)	79	0.79	10.69	104	90.20	100
[2, 3)	36	0	9.73	36	90.20	100
[3, 4)	36	0	9.73	36	90.20	100
..	..	..	..	..	..	..
[9, 10)	36	0	9.73	36	90.20	100
[15, 16)	36	0	9.73	111	87.43	97.42
[20, 21)	36	0	9.73	392	53.10	63.09
[25, 26)	36	0	9.73	332	23.81	33.80
[30, 31)	36	0	9.73	202	9.41	19.4
[35, 36)	36	0	9.73	68	0.36	10.22
[40, 41)	36	0	9.73	68	0.36	10.22
[45, 46)	36	0	9.73	36	0	9.73
..	..	..	..	..	..	..

Therefore, although the model simulates the macroscopic PTX-induced current through the Na/K-ATPase (figure 2), it describes only part of the behaviour of the single channels opened in the PTX presence (figure 3). As is shown, before the PTX perfusion, the enzyme mainly remains in the states Na^+^_3_E1 and E1. After the PTX binding, the enzyme transitions between the states PTXEc and PTXEo.

The values of the p8 constants that lead to the single channel remaining in the opened state for a relatively long time are associated with the time that the PTX stays bound to the pump, which was experimentally observed when recording the whole-cell current. On the other hand, the adjustments of the p8 constants that result in a low probability of channel aperture are associated with the PTX remaining bound for a very long time, which is not in agreement with the current decay time observed in whole-cell current recording. This difference may be due to the [PTX] used. In the case of the single-channel recordings, after the channel aperture, the PTX perfusion was discontinued. However, in the whole-cell recording, the PTX perfusion was performed with higher [PTX] during the current induction. This difference in the [PTX] resulted in different behaviours in the channel activity which may be explained by the presence of two binding sites for the toxin in the pump: one with a high affinity, which is occupied even in low [PTX] and is associated with the aperture of the single channels, and the other with a low affinity, to which the binding occurs only for high [PTX], when the opening probability of the induced channels increases.

**Figure 4.**
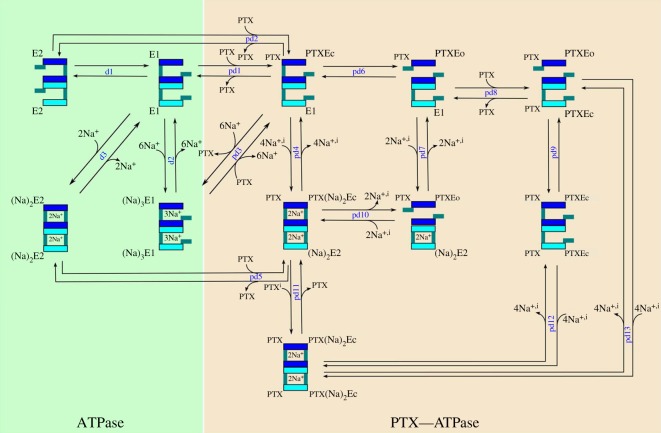
Reaction and substate diagram for the PTX–pump complex assuming the pump functions as a diprotomer.

The binding of two PTX molecules may occur only if the pump is a diprotomeric complex [14]. Evidence of the pump functioning as a diprotomer has been reported [15] and, furthermore, the experiments described above [5,6] were carried out in conditions where it is possible to consider the existence of pumps functioning as diprotomeric complexes [15]. Therefore, to reproduce and investigate the induced current dynamics in single channels, we propose a model that describes the PTX effect on the pump acting as a diprotomer (figure 4). In the model, considering only Na^+^ as the physiologic ligand, the pump substates and reactions were based on reported descriptions [15]. The following substates were assumed: (i) E1:E1—both enzymes with the intracellular gates opened; (ii) E2:E2—both enzymes with the extracellular gate opened; (iii) $N{a^{+}}_{3}E1:N{a^{+}}_{3}E1$—three Na^+^ present in the intracellular face of each enzyme; (iv) ${\left(Na^{+}\right)}_{2}E2:(Na^{+}{)}_{2}E2$—two Na^+^ occlusions in each enzyme. The reactions and corresponding reaction rates that describe the transitions between the pump diprotomer substates are shown in table 7.

**Table 7.** Reactions to describe the interactions of (Na^+^/K^+^)-ATPase with Na^+^.

index	reaction	reaction rate
d1	E2:E2 ↔E1:E1	$r_{d1}=\alpha_{1}[E2:E2]-\beta_{1}[E1:E1]$
d2	6Na^+,i^ + E1:E1 ↔${\mathrm{Na}}_{3}^{+}E1:{\mathrm{Na}}_{3}^{+}E1$	$r_{d2}=\alpha_{2}\frac{{\left({\left[Na^{+}\right]}^{i}\right)}^{6}}{{\left({\left[Na^{+}\right]}^{i}+Kd_{\mathrm{Na}}\right)}^{5}}[E1:E1]-\beta_{2}[{\mathrm{Na}}_{3}^{+}E1:{\mathrm{Na}}_{3}^{+}E1]$
d3	(Na^+^)_2_E2: (Na^+^)_2_E2↔ E1:E1 + 4Na^+,i^	$r_{d3}=\alpha_{3}[{\left(Na^{+}\right)}_{2}E2:{\left(Na^{+}\right)}_{2}E2]-\beta_{3}\frac{{\left({\left[Na^{+}\right]}^{o}\right)}^{4}}{{\left({\left[Na^{+}\right]}^{o}+Kd_{\mathrm{Na}}\right)}^{4}}[E1:E1]$

Concerning the PTX–Na^+^/K^+^-ATPase complex (figure 4 and table 8), it was assumed that low [PTX] acting on isolated induced channels is characterized by the binding of only one PTX molecule to the pump working as diprotomer. When the perfusion is performed with high [PTX], enough to induce the macroscopic current, involving a pump population, it was assumed that simultaneous binding of two PTX molecules to the pump diprotomer occurs. For the reaction of the first PTX molecule, it was assumed that PTX binds to one of the pumps of the diprotomer, in one of the substates E1:E1 (reaction pd1), E2:E2 (reaction pd2) or $N{a^{+}}_{3}E1:N{a^{+}}_{3}E1$ (reaction pd3), resulting in the substate E1:PTXEc, in which the channel is closed. From this substate, Na^+^ occlusion may occur, leading to the substate $(Na^{+}{)}_{2}E2:\mathrm{PTX}(Na^{+}{)}_{2}\mathrm{Ec}$ (reaction pd4). From the substate (Na^+^)_2_E2:PTX(Na^+^)_2_Ec, the dissociation of PTX may lead to the substate (Na^+^)_2_E2:(Na^+^)_2­_E2 (reaction pd5). The channel E1:PTXEo may be opened by a spontaneous transition from the substate E1:PTXEc (reaction pd6). The occlusion of two Na^+^ in the pump without the PTX binding to the substate E1:PTXEo leads to the substate (Na^+^)_2_E2:PTXEo (reaction pd7), which also represents an opened induced channel. The occlusion of more than two Na^+^ in the PTX–pump complex causes the closing of the induced channel (reaction pd10), forming the substate (Na^+^)_2_E2:PTX(Na^+^)_2_E2.

**Table 8.** Reactions for the PTX–ATPase complex considering the pump as a diprotomeric complex.

index	reaction	reaction rate
pd1	PTX^o^ + E1:E1 ↔ E1:PTXE_c_	$r_{\mathrm{pd}1}=\alpha_{\mathrm{pd}1}{\left[\mathrm{PTX}\right]}^{o}[E1:E1]-\beta_{\mathrm{pd}1}[E1:\mathrm{PTXEc}]$
pd2	PTX^o^ + E2:E2 ↔ E1:PTXE_c_	$r_{\mathrm{pd}2}=\alpha_{\mathrm{pd}2}{\left[\mathrm{PTX}\right]}^{o}[E2:E2]-\beta_{\mathrm{pd}2}[E1:\mathrm{PTX}E_{c}]$
pd3	$\mathrm{PT}X^{o}+{\mathrm{Na}}_{3}^{+}E1:{\mathrm{Na}}_{3}^{+}E1↔E1:\mathrm{PTX}E_{c}+6Na^{+,i}$	$r_{\mathrm{pd}3}=\alpha_{\mathrm{pd}3}{\left[\mathrm{PTX}\right]}^{o}[{\mathrm{Na}}_{3}^{+}E1:{\mathrm{Na}}_{3}^{+}E1]-\beta_{\mathrm{pd}3}[E1:\mathrm{PTX}E_{c}]\frac{{\left({\left[Na^{+}\right]}^{i}\right)}^{5}}{{\left({\left[Na^{+}\right]}^{i}+K_{\mathrm{dNa}}\right)}^{4}}$
pd4	$4Na^{+,i}+E1:\mathrm{PTXEc}\;↔{\left(Na^{+}\right)}_{2}E2:\mathrm{PTX}(Na^{+}{)}_{2}E_{c}$	$r_{\mathrm{pd}4}=\alpha_{\mathrm{pd}4}\frac{{\left({\left[Na^{+}\right]}^{i}\right)}^{4}}{{\left({\left[Na^{+}\right]}^{i}+K_{\mathrm{dNa}}\right)}^{4}}[E1:\mathrm{PTXEc}]-\beta_{\mathrm{pd}4}[{\left(Na^{+}\right)}_{2}E2:\mathrm{PTX}(Na^{+}{)}_{2}E_{c}]$
pd5	${\left(Na^{+}\right)}_{2}E2:\mathrm{PTX}(Na^{+}{)}_{2}E_{c}\;↔\;(Na^{+}{)}_{2}E2:{\left(Na^{+}\right)}_{2}E2+\mathrm{PT}X^{o}$	$r_{\mathrm{pd}5}=\alpha_{\mathrm{pd}5}[{\left(Na^{+}\right)}_{2}E2:\mathrm{PTX}(Na^{+}{)}_{2}E_{c}]-\beta_{\mathrm{pd}5}[{\left(Na^{+}\right)}_{2}E2:{\left(Na^{+}\right)}_{2}E2]{\left[\mathrm{PTX}\right]}^{o}$
pd6	E1:PTXEc ↔ E1:PTXE_o_	$r_{\mathrm{pd}6}=\alpha_{\mathrm{pd}6}[E1:\mathrm{PTXEc}]-\beta_{\mathrm{pd}6}[E1:\mathrm{PTXEo}]$
pd7	$2Na^{+,i}+E1:\mathrm{PTX}E_{o}↔{\left(Na^{+}\right)}_{2}E2:\mathrm{PTXEo}$	$r_{\mathrm{pd}7}=\alpha_{\mathrm{pd}7}\frac{{\left({\left[Na^{+}\right]}^{i}\right)}^{2}}{{\left({\left[Na^{+}\right]}^{i}+K_{\mathrm{dNa}}\right)}^{2}}[E1:\mathrm{PTX}E_{o}]-\beta_{\mathrm{pd}7}[{\left(Na^{+}\right)}_{2}E2:\mathrm{PTXEo}]$
pd8	$\mathrm{PT}X^{o}+E1:\mathrm{PTX}E_{o}↔\mathrm{PTXEc}:\mathrm{PTXEo}$	$r_{\mathrm{pd}8}=\alpha_{\mathrm{pd}8}{\left[\mathrm{PTX}\right]}^{o}[E1:\mathrm{PTXEo}]-\beta_{\mathrm{pd}8}[\mathrm{PTXEc}:\mathrm{PTXEo}]$
pd9	PTXEc:PTXEo ↔ PTXEc:PTXEc	$r_{\mathrm{pd}9}=\alpha_{\mathrm{pd}9}[\mathrm{PTXEc}:\mathrm{PTXEo}]-\beta_{\mathrm{pd}9}[\mathrm{PTXEc}:\mathrm{PTXEc}]$
pd10	$2Na^{+,i}+{\left(Na^{+}\right)}_{2}E2:\mathrm{PTX}E_{o\;}↔{\left(Na^{+}\right)}_{2}E2:\mathrm{PTX}(Na^{+}{)}_{2}\mathrm{Ec}$	$r_{\mathrm{pd}10}=\alpha_{\mathrm{pd}10}\frac{{\left({\left[Na^{+}\right]}^{i}\right)}^{2}}{{\left({\left[Na^{+}\right]}^{i}+K_{\mathrm{dNa}}\right)}^{2}}[{\left(Na^{+}\right)}_{2}E2:\mathrm{PTXEo}]-\beta_{\mathrm{pd}10}[{\left(Na^{+}\right)}_{2}E2:\mathrm{PTX}(Na^{+}{)}_{2}\mathrm{Ec}]$
pd11	$\mathrm{PT}X^{o}+{\left(Na^{+}\right)}_{2}E2:\mathrm{PTX}E_{o\;}↔\mathrm{PTX}(Na^{+}{)}_{2}\mathrm{Ec}:\mathrm{PTX}(Na^{+}{)}_{2}\mathrm{Ec}$	$r_{\mathrm{pd}11}=\alpha_{\mathrm{pd}11}{\left[\mathrm{PTX}\right]}^{o}[{\left(Na^{+}\right)}_{2}E2:\mathrm{PTXEo}]-\beta_{\mathrm{pd}11}[\mathrm{PTX}(Na^{+}{)}_{2}\mathrm{Ec}:\mathrm{PTX}(Na^{+}{)}_{2}\mathrm{Ec}]$
pd12	$4Na^{+,i}+\mathrm{PTXEc}:\mathrm{PTXEc}↔\mathrm{PTX}(Na^{+}{)}_{2}\mathrm{Ec}:\mathrm{PTX}(Na^{+}{)}_{2}\mathrm{Ec}$	$r_{\mathrm{pd}12}=\alpha_{\mathrm{pd}12}\frac{{\left({\left[Na^{+}\right]}^{i}\right)}^{4}}{{\left({\left[Na^{+}\right]}^{i}+K_{\mathrm{dNa}}\right)}^{4}}[\mathrm{PTXEc}:\mathrm{PTXEc}]-\beta_{\mathrm{pd}12}[\mathrm{PTX}(Na^{+}{)}_{2}\mathrm{Ec}:\mathrm{PTX}(Na^{+}{)}_{2}\mathrm{Ec}]$
pd13	$4Na^{+,i}+\mathrm{PTXEc}:\mathrm{PTXEo}↔\mathrm{PTX}(Na^{+}{)}_{2}\mathrm{Ec}:\mathrm{PTX}(Na^{+}{)}_{2}\mathrm{Ec}$	$r_{\mathrm{pd}13}=\alpha_{\mathrm{pd}13}\frac{{\left({\left[Na^{+}\right]}^{i}\right)}^{4}}{{\left({\left[Na^{+}\right]}^{i}+K_{\mathrm{dNa}}\right)}^{4}}[\mathrm{PTXEc}:\mathrm{PTXEo}]-\beta_{\mathrm{pd}13}[\mathrm{PTX}(Na^{+}{)}_{2}\mathrm{Ec}:\mathrm{PTX}(Na^{+}{)}_{2}\mathrm{Ec}]$

The binding of the second PTX molecule to the pump in the substate E1:PTXEo leads to the substate PTXEc:PTXEo (reaction pd8). From the substate PTXEc:PTXEo, the channel may close by means of a spontaneous transition (reaction pd9), forming the substate PTXEc:PTXEc, or by means of Na^+^ occlusion (reaction pd13), resulting in PTX(Na^+^)_2_Ec:PTX(Na^+^)_2_Ec. The following reactions were also considered: pd11—binding of the second PTX molecule to pumps in the substate (Na^+^)_2_E2:PTX(Na^+^)_2_Ec; and pd12—Na^+^ occlusion in channels in the substate PTXEc:PTXEc. As can be observed, we are not considering substates where the PTX–Na^+^/K^+^-ATPase complex exhibits two simultaneous channel apertures in a single diprotomer. This justifies the linear dependence of the activation rate of the induced current on [PTX]^o^ [4]. Therefore, only one of the channels must be induced, even when two PTX molecules are bound to the diprotomer [15].

Parameter values used in the simulations are presented in table 9. To describe the dynamic of the PTX-induced channels, the values of several constants (table 7) were considered, with values extracted from MDE [9], which were adjusted to describe the dynamic of the macroscopic current. Therefore, only eight parameters were adjusted for the present model. Constants of the reactions pd6 (*α*_pd6_ and *β*_pd6_), pd7 (*α*_pd7_ and *β*_pd7_) and pd10 (*α*_pd10_ and *β*_pd10_) were adjusted to represent the dynamic of a single PTX-induced channel. Two additional parameters, *α*_pd12_ and *β*_pd13_, were estimated to reproduce macroscopic current decay after stopping the PTX perfusion.

**Table 9.** Reactions for the PTX–ATPase complex considering the pump as a diprotomeric complex (tables 5 and 6).

		constants	
index	reference	*α_j_*	*β_j_*
d1	*α*_d1_ = *α*_1_, *β*_d1_ = *β*_1_	1.00 × 10^2^ s^−1^	1.00 × 10^−2^ s^−1^
d2	*α*_d2_ =* α*_2_, *β*_d2_ = *β*_2_	2.00 × 10^2^ mM^−1^ s^−1^	8.00 × 10^2^ s^−1^
d3	*α*_d3_ =* α*_3_, *β*_d3_ = *β*_3_	5.00 × 10^1^ s^−1^	1.00 × 10^−1^ s^−1^
pd1	*α*_pd1_ =* α*_p1_, *β*_pd1_ = *β*_p1_	3.23 × 10^3^ mM^−1^ s^−1^	1.66 × 10^−5^ s^−1^
pd2	*α*_pd2_ =* α*_p2_, *β*_pd2_ = *β*_p2_	4.30 × 10^2^ mM^−1^ s^−1^	2.20 × 10^−10^ s^−1^
pd3	*α*_pd3_ =* α*_p3_, *β*_pd3_ = *β*_p3_	5.15 × 10^2^ mM^−1^ s^−1^	6.60 × 10^−7^ mM^−1^ s^−1^
pd4	*α*_pd4_ =* β*_p5_, *β*_pd4_ = *α*_p5_	1.31 × 10^−4^ s^−1^	1.70 × 10^−4^ s^−1^
pd5	*α*_pd5_ =* α*_p5_, *β*_pd5_ = *β*_p6_	8.80 × 10^−5^ s^−1^	2.65 × 10^1^ mM^−1^ s^−1^
pd6	adjusted	1.33 s^−1^	5.09 s^−1^
pd7	adjusted	5.00 × 10^−3^ s^−1^	2.50 × 10^−4^ s^−1^
pd8	*α*_pd8_ =* α*_p1_, *β*_pd8_ adjusted	3.23 × 10^3^ mM^−1^ s^−1^	1.66 × 10^−4^ s^−1^
pd9	*α*_pd9_ =* β*_p6_, *β*_pd9_ = *α*_p6_	1.33 s^−1^	5.09 s^−1^
pd10	adjusted	1.33 × 10^1^ s^−1^	1.33 × 10^−1^ s^−1^
pd11	*α*_pd11_ =* α*_pd5_, *β*_pd11_ = *β*_pd5_	8.80 × 10^−5^ s^−1^	2.65 × 10^1^ mM^−1^ s^−1^
pd12	*α*_pd12_ adjusted, *β*_pd12_ = *β*_pd11_*α*_pd12_*α*_pd9_*β*_pd8_*β*_pd4_*α*_pd6_/	1.70 × 10^−4^ s^−1^	1.39 × 10^−6^ s^−1^
	*α*_pd11_*β*_pd9_*α*_pd8_*α*_pd4_*β*_pd6_		
pd13	*α*_pd13_ = *β*_pd13_*α*_pd12_*α*_pd9_ /*β*_pd9_*β*_pd12_, *β*_pd13_ adjusted	4.20 × 10^−4^ s^−1^	1. 31 × 10^−5^ s^−1^

**Figure 5.**
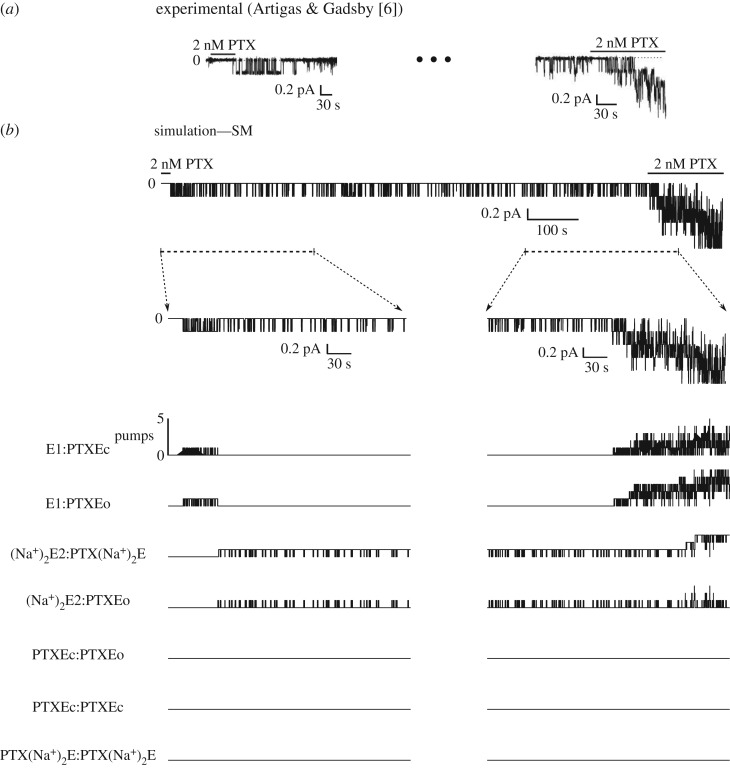
Single-channel activity and activity of a few channels induced by the presence of PTX. (*a*) The perfusion with solution containing PTX (2 nM) caused the channel to open, showing several open/close transitions and epochs where the channel remains in the closed state most of the time. After a few minutes of channel activity, the perfusion with PTX (2 nM) continues, causing the appearance of more channel activity. (*b*) Simulation of the induced channel activity. Reproducing the experimental manoeuvre, the [PTX]o was maintained at 2 nM, until the channel opened, therefore, until the pump occupies the substate PTXEo. After the channel remains active for a few minutes, the [PTX]o is changed from 0 to 2 nM, inducing more channels. (Left) The period of most intense activity is characterized by transitions between the substates E1:PTXEc and E1:PTXEo (p6 reaction). In the period where the activity is less intense, the pump occupies the substates (Na^+^)2E2:PTXEo and (Na^+^)2E2:PTX(Na^+^)2Ec. (Right) The continuous perfusion with PTX induces channels occupying all substates E1:PTXEc and E1:PTXEo.

Simulating the continuous perfusion of PTX until the first channel aperture (figure 5), it can be seen that assuming the pump is a diprotomer allows for reproducing the behaviour of the PTX-induced current in isolated channels, in accordance with experimental recordings [6]. The current contains epochs with intense activity and epochs where the activity is reduced, remaining almost in the closed state (zero current). In epochs where the activity is intense, the PTX–Na^+^/K^+^-ATPase complex is mainly in the substates E1:PTXEc and E1:PTXEo. The activity reduction occurs when the PTX–Na^+^/K^+^-ATPase complex is in the substates with Na^+^ occlusion: (Na^+^)_2_E2:PTX(Na^+^)_2_E and (Na^+^)_2_E2:PTXEo. The substates with two PTX molecules bound to the pump are not occupied, indicating a low probability of occurrence due to the [PTX]^o^ used in the perfusion solution. As in the experiments, after minutes of activity in the PTX-induced channel, the reperfusion of PTX (2 nM) causes a progressive induction of new channels. Again, the perfusion with low [PTX] induces channels mainly occupying the substates E1:PTXEc, E1:PTXEo, (Na^+^)_2_E2:PTX(Na^+^)_2_E and (Na^+^)_2_E2:PTXEo. No binding of the second PTX molecule is present, demonstrated by the absence of substates with two PTX molecules bound to the pump.

**Figure 6.**
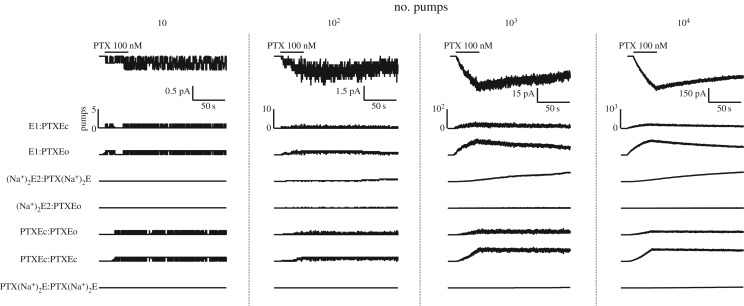
Each column shows a simulation with a different number of pumps (top traces). The corresponding PTX-induced current (below). The number of pumps occupying the different substates is considered in the model.

By increasing [PTX] (100 nM), it is possible to simulate the activity of a few channels or the macroscopic current induced by the toxin (figure 6). When a few pumps (10 or 100) are observed during the PTX perfusion, the channel activity is more evident. In these situations, the pumps bound by PTX mainly occupy the substates without Na^+^ occlusion. When the number of available pumps is increased (10^3^ or 10^4^ pumps), the simulation shows the macroscopic induced current. In this case, not only are the substates without Na^+^ occlusion present; the occlusion of Na^+^ results in the formation of the substate (Na^+^)_2_E2:PTX(Na^+^)_2_Ec. The activation rate of the macroscopic current induced by PTX (10^4^ pumps) for the induced channel functioning as a diprotomer (figure 6) was slower (1/*τ* = 0.02 s^−1^) than that adjusted for the model of the channel functioning as a monomer (figure 2; 1/*τ* = 0.07 s^−1^). However, this slower rate is also within the experimentally recorded range, in the absence of nucleotides and when the pump is perfused only with solution containing the physiological ligands [4–6].

## 4. Discussion

The goal of this work is to investigate, using an SM, the substates of the Na^+^/K^+^ pump associated with a PTX-induced current, assuming the exclusive presence of Na^+^ acting as a physiological ligand of the pump in the intra- and extracellular spaces. Initially, the SM was derived from a reaction model [9], assuming the pump is a monomeric complex. However, although the SM allows for simulating the macroscopic current induced by PTX, it was not possible to reproduce the observed behaviours of the current induced by the toxin through a single channel. The study shows that if we assume that the Na^+^/K^+^ pump-induced channel functions as a diprotomer, it is possible to simulate the dynamic of the current induced by PTX acting on the Na^+^/K^+^ pump through isolated channels and for the channel population. However, the simulations do not exclude the possible coexistence of pumps functioning in both conditions, monomeric and diprotomeric complexes, in the same cellular membrane.

The UPPAAL-SMC platform was useful for fast adjustment of the parameters of the model. As described in tables 5 and 6, the probabilistic information based on the statistical model checker generated the stochastic semantics and provided a reduced number of simulations and, at the same time, offered simulations that better represent the average behaviour and better interpret the toxin effects.

The proposed model, implemented in UPPAAL-SMC, can be used as a profitable tool to guide and/or analyse experimental procedures for recording single channels in different ionic concentration conditions for Na^+^. This may contribute to improving the model and, consequently, to comprehending the kinetics of PTX-induced channels. For the pump functioning in the absence of PTX and considering Na^+^ as the sole physiological ligand, the proposed model was based on the kinetics of the substate transitions described in the Albers–Post model. Moreover, the substates that may be occupied by the diprotomeric complex were represented according to reported hypotheses [15]. The substates and substate transitions of the PTX–Na^+^/K^+^-ATPase complex were proposed following a reported kinetic model [9] and, therefore, some constants were the same (table 9). The parameters of the reactions were directly related to the channel induction and its activity and, consequently, with the induced current, and were adjusted based on the current due to a few ionic channels [5,6]. Moreover, validating the performed adjustments, it was possible to reproduce the dynamic of several channels (figures 5 and 6). It can be observed that the macroscopic current activation rate after the adjustments was smaller (figure 6) than that before adjustments when considering the pump functioning as a monomer (figure 2). However, this value is still within the experimental range [4–6].

### 4.1. Palytoxin interactions with the Na^+^/K^+^-ATPase: evidence of the pump functioning as a diprotomer

Considering the pump as a monomeric complex and using the SM, it was possible to simulate the experimental behaviour of the current induced with PTX perfusion in the presence of Na^+^ in the intra- and extracellular spaces (figure 2), as experimentally recorded [5,6]. Furthermore, it was also possible to reproduce the behaviour of the substates of the PTX–Na^+^/K^+^ pump complex responsible for the toxin-induced current, which were also simulated using a model based on differential equations (MDE) that describes the PTX effect on the Na/K-ATPase enzyme [9]. In this study, the SM was developed based on the same MDE, including only one more equation involving the PTX–Na^+^/K^+^-ATPase complex: reaction p8. This equation describes the opening and the closing of the PTX-induced channel, causing transitions between the substates PTXEc and PTXEo. The aperture of the channels induced by the toxins was described in the MDE [9] and assumes that the normalized permeability of the substrate PTXE is equal to 0.19, which is the value of the opening probability of the channels in the absence of ATP [6]. In the SM, the constants *α*_p8_ and *α*_p8_ were adjusted to describe the activation of the macroscopic current induced by PTX (figure 2). With these adjustments, it was possible to reproduce the opening probability of a single channel (approx. 0.19). However, in the experiments recording a single channel induced by PTX [5,6], it can be seen that in the absence of ATP, the channel activity exhibited epochs with relatively high opening probability followed by long epochs of low opening probability where the channel remains in the closed state for longer, despite the presence of the toxin (figure 3). This observation indicates the possible existence of different conformational substates of the PTX–Na^+^/K^+^-ATPase complex that represent the opened channels induced by the toxin, even in the absence of ATP. Supporting this hypothesis, it was not possible to reproduce the behaviour of the single channels induced by PTX when the SM represented the pump as a monomeric complex (figure 3). It was possible to simulate solely the epoch with a high opening probability or, readjusting the constants of the reaction p8, the epoch where the channel remains in the closed state for a longer period.

The existence of an additional substate of the PTX-induced channels can be justified if the Na/K pump works as a diprotomer in the presence of the toxin. There is evidence that the Na/K pump should function as an oligomeric complex [15–17]. In the diprotomer complex (*αβ*)_2_, a cooperative positive feedback between the proteins increases the affinity for the binding of the first ATP molecule. Therefore, in very low concentrations of ATP, this nucleotide may bind more effectively to the pump and, consequently, result in an ionic transport rate greater than when functioning as a monomeric complex, a situation where the ATP affinity is reduced. Moreover, the second ATP molecule binding to the diprotomer exhibits a lower affinity (approx. 40 times less intense), causing the dissociation of the diprotomer into individual monomers and increasing the phosphorylation rate of the Na^+^/K^+^-ATPase [15].

In this work, the SM considers the pump channel to be a diprotomer (figure 4) and allows for reproducing the behaviour of the single channels induced by PTX and the macroscopic current generated by a channel population (figures 5 and 6). The binding of the first PTX molecule to the diprotomeric complex has higher affinity than the second molecule binding (*β*_pd1_/*α*_pd1_ < *β*_pd8_/*α*_pd8_). Therefore, the cooperative protein–protein effect in the diprotomer, which is positive in the case of the ATP binding [14], may be interpreted as negative for the PTX binding. This allows PTX to bind to the pump even at very low concentrations, changing its function and inducing the channels, making its toxic effect more effective.

The most accepted hypothesis of the formation of the Na/K pump channels induced with PTX suggests that this formed channel includes the path for the transport of Na^+^ and K^+^ by the pump[4–6]. Although not excluding this hypothesis, the simulations presented in this study also suggest an alternative hypothesis: PTX binding to the pump may stabilize the diprotomeric structure of the pump, resulting in a channel permeable to monovalent ions. This channel may not be directly involved in the transporting paths of Na^+^ and K^+^ through the pump structure. The channel may be formed by the molecular chain created by the diprotomer formation and stabilized by PTX.

Additionally, the model proposed in the present work suggests that the channel induced when bound to a single PTX molecule presents two gating modes, defined by different levels of activity between which the channel may transition instantaneously [18]. One gating mode is described by the transition between the states E1:PTXEc and E1:PTXEo (reaction pd6), characterizing a high level of channel activity with a relatively high value for the opening channel probability. The second gating mode is represented by the reaction pd10, which is described as a transition between the states (Na^+^)_2_E1:PTXEo and (Na^+^)_2_E1:PTX(Na^+^)_2_E. This second gating mode can represent a low opening probability rate and characterizes a quiescent state, which is interpreted as an interval between bursts [18]. In the model, the transition between these two gating modes is caused by the Na^+^ occlusion in the *α* chain of the diprotomer, which is bound to PTX (reactions pd4 and pd7). As can be observed in table 9, the transitions between substates of the same gating modes (reactions pd6 and pd10) are faster than the transitions between substates of different gating modes (reactions pd4 and pd7): *α*_pd6_, *β*_pd6_, *α*_pd10_ and *β*_pd10_ are greater than *α*_pd4_, *β*_pd4_, *α*_pd7_ and *β*_pd7_. This is an expected behaviour because, by definition, transitions between gating modes are observed on a slower time scale [18].

### 4.2. Na^+^ interactions with the PTX–Na^+^/K^+^-ATPase complex

Na^+^ occlusion affects the opening probability of the PTX-induced channels due to this ion promoting the closure of the pump gates [9]. In addition, this study suggests that Na^+^ occlusion in the α chain without PTX binding (pd7 reaction) also affects the opening probability of the channels. This occurs because the opening–closing transitions that were taking place between the substates E1:PTXEc and E1:PTXEo by means of reaction pd6 became governed by the substates (Na^+^)_2_E2:PTX(Na^+^)_2_Ec and (Na^+^)_2_E2:PTXEo (reaction pd10). According to the simulations (figure 5), the transitions due to reaction p6 characterize the highest activity of the induced channel, which were recorded experimentally in a single-channel configuration [5,6], with an open state probability estimated at approximately 0.19%. The transitions by means of the reaction p10 are responsible for the long periods where the channel remains closed. In addition, due to the Na^+^ occlusion/deocclusion in the channel not bound by PTX, reaction p7 is responsible for the transitions between high- and low-level activities in the PTX-induced channels. The effect of the Na^+^ binding to an α chain of the diprotomer was also reported [14]. The author observed that the presence of three Na^+^ in one α chain of the pump, forming the substate (K^+^)_2_E2:Na^+^_3_ E1, reduces the transition rate of the second protomer to state E1. The reaction (K^+^)_2_E2(K^+^)_2_ E2→(K^+^)_2_E2:(Na^+^)_3_ E1 is faster than the reaction (K^+^)_2_E2(Na^+^)_3_ E1→(Na^+^)_3_E1:(Na^+^)_3_ E1 [14].

As shown in table 9, the microscopic reversibility principle was used for the determination of the parameters *β*_pd12_ and *α*_pd13_, reducing the degree of freedom during the parameter adjustment process. Only the closed cycle performed from the pd6, pd7, pd10 and pd4 reactions did not allow satisfying this principle when the parameters of the model were adjusted. The following possibilities emerge from this observation: (i) the microscopic reversibility principle is violated in this situation or (ii) there must be at least one more intermediate substate in this open/close cycle. The former is possible because the current through the induced channels was generated by means of a Na^+^ gradient and transmembrane potential and the Na^+^ flux is also dependent on the opening and closing of the channel. These are conditions where the reversibility principle may be violated even when the channel activities are in the equilibrium state [19–21]. With respect to the second hypothesis, the existence of an intermediate substate between (Na^+^)_2_E2:PTX(Na^+^)_2_Ec and (Na^+^)_2_E2:PTXEo, such as (Na^+^)_2_E2:PTX-Na^+^_2_Ec, can be proposed, indicating that reaction pd10 would be redefined as (Na^+^)_2_E2:PTX-Na^+^_2_Ec↔ (Na^+^)_2_E2:PTXEo, with the same values adjusted for the constants and a new reaction would be incorporated: (Na^+^)_2_E2:PTX(Na^+^)_2_Ec ↔ (Na^+^)_2_E2:PTX-Na^+^_2_Ec (reaction pd14). Assuming no violation of the reversibility principle, then *β*_pd14_ = 1 × 10^−4^ × *α*_pd14_. Since reaction pd14 would involve only the transition between substates in which the channels are closed, additional experimental data with favourable conditions would be necessary to determine the value of the constant *α*_pd14_.

## 5. Conclusion

In this study, we proposed a new methodology to extract information about state transitions and the corresponding reactions of ionic channels. For PTX-induced channels, we used an SM implemented in UPAAL-SMC to investigate the behaviour of ionic currents recorded from a single channel and a population of channels by means of probabilistic analyses and simulations. The reproduction of PTX-induced currents (single channel and population of channels) through the Na^+^/K^+^ pump, as performed in this study, provided support for the hypothesis that this pump may act as a diprotomer (*αβ*)_2_, able to be bound by two PTX molecules. The cooperative effect of the protomers is negative because the affinity to PTX is increased and, therefore, the toxicity is more effective even in lower concentrations of the toxin. Moreover, the simulations suggest that Na^+^ may modulate the opening and closing of the isolated channels, reducing the activity, as observed in single-channel current recordings.

## Data accessibility

The UPPAAL (v. 4.1.19 for Linux—academic licence) was obtained from http://www.uppaal.org/. The source codes implemented in UPPAAL are deposited at the Dryad Digital Repository: http://dx.doi.org/10.5061/dryad.qn254 [22].
